# Oxidative Stress and Space Biology: An Organ-Based Approach

**DOI:** 10.3390/ijms19040959

**Published:** 2018-03-23

**Authors:** Thomas J. Goodwin, Melpo Christofidou-Solomidou

**Affiliations:** 1 The National Aeronautics and Space Administration (NASA, retired) Johnson Space Center, Houston, TX 77058, USA; 2 Division of Pulmonary, Allergy, and Critical Care Medicine and the Department of Medicine, University of Pennsylvania Perelman School of Medicine, 3450 Hamilton Walk, Edward J. Stemmler Hall, 2nd Floor, Office Suite 227, Philadelphia, PA 19104, USA

## 1. Introduction

The environment of space provides many challenges to the human physiology and therefore to extended habitation and exploration. Research to define and countermeasures to address these challenges have been explored for well over 40 years yet success in alleviating these challenges has been inadequate, due in part to the lack of investigative tools (subject number and technology) to monitor and diagnose conditions in real time and by the overall lack of a relevant data base to understand the central causative agent(s) responsible for physiological adaptation and homeostasis in space [1,2,3,4,5]. Space flight adaptation syndromes are a product of the environmental conditions and the synergistic reaction of the systemic human physiology, which together combine to produce a combinatorial syndrome that must be resolved in order to safely inhabit and explore space, especially for extended periods [3,4,5,6]. This paper documents an exciting glimpse of current pertinent scientific literature sustaining the involvement of oxidative stress and damage (OSaD) [7,8] as a significant contributing factor in the following areas of Earth-based and space flight-related dysregulation of: (1) bone loss [9,10,11,12,13]; (2) cardiovascular function [14]; (3) immune insufficiency and metabolism [15,16,17]; (4) neurological impairment [18]; and (5) potential countermeasure implementation [18,19]. The cited literature illuminates the environmental challenges of spaceflight encompassing reduced gravity, radiation, varying atmospheric conditions, such as hyperoxia and hypoxia experienced during extravehicular and intravehicular activity (EVA/IVA), and evidence of synergistic effects that portend substantial consequences for long duration/exploration class missions. Now with 21st century scientific tools and advances in genomics, proteomics, and metabolomics (“omics”) we have the resources to define syndromes at the molecular and systemic levels by enlisting a holistic approach to the assessment of the space flight physiology and to countermeasure development. To accomplish this, one must seek a “common denominator” that has systemic effects on the function of the entire physiology and stems from both environmental conditions (reduced gravity, radiation, mental and physical stress, etc.) and the byproducts of the human systems biology (reduction oxidation (REDOX)) equilibrium, free radical balance, and reactive oxygen and nitrogen species (ROS and RNS). One such common denominator is the stabilization of physiological homeostasis and control of OSaD in the body [7,8,20]. 

REDOX equilibrium is cyclic biofeedback related biochemistry. OSaD control/REDOX homeostasis encompasses many aspects of the human physiological response to the space environment and if mitigated properly may normalize a portion of the adverse phenomena seen in space flight combinatorial events and suggest relevant countermeasures for those situations, thus increasing the level of safety and occupational health for each crew member during long duration flight and facilitate improved performance during exploration class missions. 

## 2. Overview

The generation of ROS/RNS in the human physiology is a normal part of human systems biology [7,8]. Oxidative stress and damage (OSaD) is, however, the result of organic and systemic dysregulation of the free radical normalization and scavenging process. Documented sources of OSaD encompass a range of factors which can be divided into two broad categories, being those comprised of physiological and environmental conditions [7,8]. They include, but are not limited to, physiological factors such as physical and psychological stress, poor or constrained diet and nutrition, exercise and/or lack thereof, immune dysregulation and malfunction (autoimmune and wasting syndromes etc.), cardiovascular insufficiency, endocrine imbalances, genotype, and cancers. In addition, environmental conditions such as radiation exposure (occupational exposure, cancer therapy), cancer chemotherapy (toxicity/poisons), pharmaceutical use, exposure to hypoxia or hyperoxia (industrial divers and pilots, etc.), allergens and environmental pollutants all play significant roles in the assault on the human body’s ability to equilibrate the REDOX coefficient systematically, in short to control OSaD. Thus, OSaD is the result of loss of homeostasis in the human physiology regarding the body’s REDOX equilibrium [7,8]. These conditions have been documented in the scientific literature and some of that literature is provided herein.

**Figure 1:**
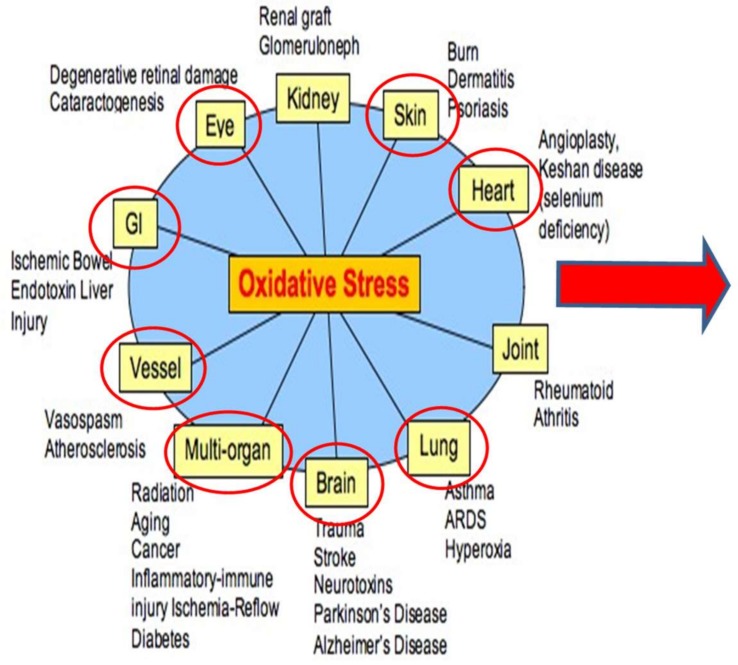
This graphic depiction of human disease states linked to Oxidative Stress and Damage (OSaD) reflects the categories and similarities, although to a lesser degree, experienced by astronauts. Adapted from National Institute of Standards and Technology (NIST)^6^.

Space travel and persistent habitation of reduced gravity or non-terrestrial environments poses many problems which are in some respects similar to the systems biology of terrestrial living associated with numerous disease states and the progression of aging (Figure 1) [6,7]. However, counter to the avenues and facilities available to humans in 1G, astronauts, although they are generally very healthy, are confined to remarkably smaller volumes, with restricted access to environmental, medical, and recreational facilities. Thus, as we begin to prepare for exploration beyond low Earth orbit (BLEO), it is obligatory to assess the similarities in OSaD loads on Earth to those in space and provide reasonable countermeasures to these imbalances. Furthermore, in the exploration environment, astronauts are and will be faced with a host of simultaneous events, again unlike the average human terrestrial resident. 

**Figure 2:**
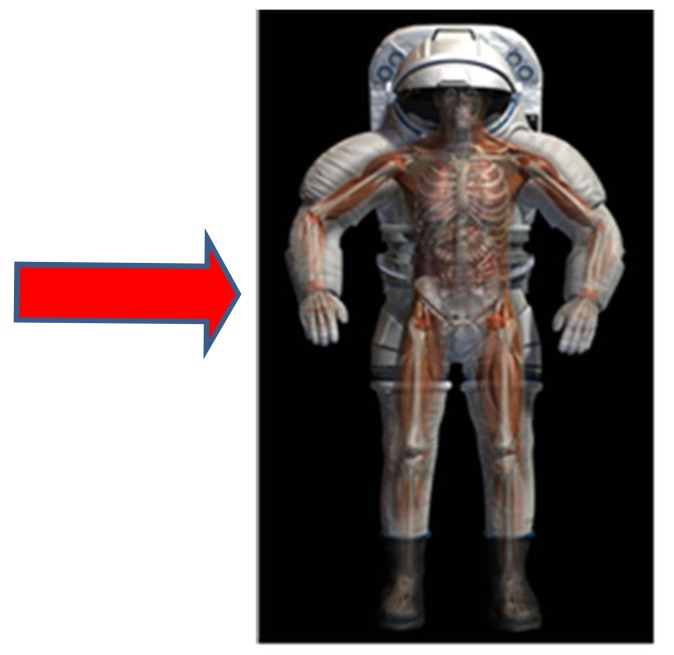
This illustration demonstrates the general construct of the space suit and the lack of substantive shielding leading to increased radiation exposure and generation of OSaD. Courtesy of NASA Johnson Space Center.

This Special Issue of IJMS presents some recent supporting data and possible countermeasure approaches. Figure 1 and Figure 2 serves to illustrate the complex myriad of influences on OSaD production and REDOX imbalance which affect the physiology and are central to the theme of this research. The graphic below demonstrates the impact of both environmental and physiological factors that come together to mediate the relationship of OSaD to disease and loss of homeostatic control.

Most of the conditions exemplified in Figure 1 and Figure 2 are relevant to space flight, especially those influencing eye, brain, bone, vessel, heart, lung and other multi-organ systems. The figure illustrates the broad reaching influence of OSaD in the human system and thereby the relevance to space habitation. Calculations and accurate assessment of the potential “space hazards” for human survival require the composite expression of multiple factors that present in the space environment, as seen in the figure above, rather than an isolated single upset event approach. 

Mammalian, specifically human systems biology depends on a complicated and somewhat fragile series of dynamic checks and balances to constantly provide homeostasis. An essential part of this regulatory process is the production and scavenging of excess ROS and RNS to maintain an appropriate balance of free radicals (FRs) in the physiology. Some of the FR species particularly important for space physiology are nitric oxide (NO) as it has profound cardiovascular and sub-cellar metabolic impacts, hydrogen peroxide (H_2_O_2_) as it via Haber-Weiss reaction interacts with ferric and other ions reduced by superoxide radicals [7]. This is particularly important as astronauts tend to have high iron content through plasma loss and food content in their blood and Peroxynitrite (–ONOOH) as a downstream molecule capable of depleting sulfhydryl groups. All these represent species responsible for lipid peroxidation, fatty acid loss, protein degradation and DNA damage [2,5,9,20]. Loss of this competency or genetic insufficiency or both normally results in sickness, disease, and eventual death if not resolved. 

## 3. Special Issue Manuscripts

In this Special Issue, a total of 10 excellent papers consisting of six original research studies and four reviews have been published, as detailed in Table 1.

As initially outlined above, the effects of OSaD are evident in almost all physiological organ subsystems. Here we present the some of the current literature in the field. Published manuscripts are grouped according to the specific organ subsystems affected but also show to a considerable extent the overlapping interactions. This aspect of the research field exemplifies the need for a “common denominator” approach to understanding and treating these physiological effects.

Increased radiation exposure is one of the largest challenges to the prolonged habitation of space, generating substantial amounts of ROS and RNS species. While the increase in radiation exposure in low Earth Orbit is manageable due to the protection of the Van Allen belts, part of the Earth’s protective electromagnetic field, it is by no means inconsequential. Each individual astronaut, depending on age, is allowed a defined term in space, thus limiting the amount of time one can fly. Alternatively, prolonged stays in space such as in the International Space Station (ISS) are limited normally to two 6-month tours [3,4,5]. This restriction is in place in part due to the manner in which radiation affects the bone and muscle. 

Alwood et al. observed the effects of radiation on the process of osteoblastogenesis by studying the skeletal structure of C57BL6J mice exposed to either low LET (low energy transfer) protons (50 cGy) or high LET ^56^Fe ion radiation [9]. They suggest that low energy did not induce significant cellular responses, however the high LET ^56^Fe ions (200 cGy) were responsible for increases in marrow cells production of mineralized nodules ex vivo regardless of radiation type or dose; and ^56^Fe (200 cGy) inhibited osteoblastogenesis by more than 90% (5 weeks and 1 year post-IR). After 5 weeks, irradiation (protons or ^56^Fe) resulted in minimal changes in gene expression levels during osteoblastogenesis, however a high dose ^56^Fe (200 cGy) resulted in bone loss, increased Catalase and Gadd45 gene expression, and the radiation damage seemed to be mitigated by the additions of superoxide dismutase (SOD) [10].

In a simulated microgravity culture system (RWV) Shanmugarajan et al. investigated the combined effects of gamma radiation and microgravity by monitoring the maturation of a hematopoietic cell line RAW 264.7 into mature osteoclasts. In short, results of the investigation demonstrated radiation alone at 100 cGy may stimulate osteoclast cell fusion determined via giant multinucleated cells GMCs presence and the expression of the signature genes tartrate resistant acid phosphatase (Trap) and dendritic cell-specific transmembrane protein (Dcstamp). Notably, in 1G controls osteoclast cell fusion decreased in doses above 0.5 Gy. By comparison to radiation exposure, simulated microgravity resulted in increased levels of cell fusion, and the effects of radiation and microgravity are additive. Remarkably, simulated microgravity culture effects on osteoclast stimulatory transmembrane protein (Ocstamp) and Dcstamp expression was substantially higher than the radiation effect, inferring that microgravity may increase the synthesis of adhesion molecules more than radiation [11] and thus, demonstrating the interlocking effects of the multiple environmental factors seen in space.

In light of the excellent work by Alwood et al. and Shanmugarajan et al. [10,11] it would seem prudent to consider a holistic approach to ROS and RNS countermeasures. In their review, Tian et al. suggest that the consumption of certain vitamin supplements (e.g., vitamins C and E and carotenoids) may reduce OSaD in bones and, additionally, consuming a diet high in naturally occurring antioxidants, such as carotenoids and flavonoids, may have the ability to mitigate microgravity-induced skeletal involution. Further, natural agents curcumin and turmeric, mayshow promise to attenuate hind-limb unloading (HLU)-induced bone loss by suppressing oxidative stress. Other natural products, many which are under consideration by NASA, have demonstrated skeletal benefits against OSaD. Tanshinol, rescued the decrease of osteoblastic differentiation via down-regulation of FoxO3a signaling and upregulation of Wnt signal under oxidative stress. Further antioxidants, like lipoic acid and *N*-acetyl cysteine, show promise in the arrest of oxidative stress in bone as well. Though these are reports from Earth-based studies, still they suggest important avenues to be pursued in the space flight arena to combat OSaD during prolonged flight [12]. 

In their review, Tahimic and Globus intertwine another important facet of space flight and the complex environment that consists of changes in the general physiology including bone, muscle and the vascular system; all subject to systemic OSaD effects. Microgravity forces selective environmental pressures, causing a cephalad fluid shift, profound reductions in mechanical loading of bone and muscle, and a reduced immune competency concomitant with inflammation, coupled with galactic cosmic radiation (GCR) and high energy particles (HZE). Here the authors review spaceflight-induced perturbations in calcium homeostasis and specific physiological reductions in bone mass (osteopenia), which pose protracted risks for tissue repair and skeletal health. Space analogue rodent models for these types of studies such as hind-limb unloading (HLU) result in not only bone but also vascular anomalies reminiscent of aging like vascular density (rarefication), and vasodilation responses. These responses tied to excess ROS/RNS, (NO) and inflammation are implicated both in diseases of aging, like osteoporosis and atherosclerosis, and pursuant to insults such as radiation exposure as seen in spaceflight. ROS can directly facilitate bone osteoclastogenesis, leading to resorption and bone loss during aging and estrogen deficiency may be partly attributed to OSaD mechanisms, and thus portends to link the complex phenomena of bone and muscle loss, vascular deconditioning, and immune functions together in an intricate syndrome [13]. 

To address the cardiovascular system as related to ROS, Takahasi et al. reviewed the effects of OSaD on the cardiovascular system in the presence of reduced gravity environments the central constituents being microgravity and radiation. Prior research has shown that 3–4-week HLU resulted in increased superoxide anion levels in the carotid and adjacent arteries of rats. In this investigation, eNOS expression was upregulated in the carotid artery. The effect of microgravity on the cardiovascular system seems to be different depending on the region. This is not necessarily surprising due to the graded simulation of HLU. Longer HLU studies presented increased superoxide levels, elevation of pro-oxidative enzymes NOX2 and NOX4 and reduction of important anti-oxidative enzymes like Mn-SOD and GPx-1, an effect not seen in mesenteric arteries. Once again, the inflight studies demonstrate synergistic ROS effects by the combination of radiation and microgravity. In other HLU-7-day studies on mouse brains, low dose radiation (LDR) and HLU evidenced lipid oxidation in the brain cortex, but in neither of the independent conditions alone. In 9-month studies of ROS effects, lipid peroxidation and expression of NOX2 were seen in both LDR and HLU yet the combinatorial effect was still greater [14]. 

Thus, it would appear that the physiology may be resistant to short term single event upset and thereby conceal larger problems in evidence when model studies incorporate multiple stressors as seen in actual microgravity.

We see a myriad of factors impinge on the human physiology in space. Microgravity and increased radiation are environmental catalysts for very complex systemic responses driven to abate a cascade of negative events realized singularly in space. For example, at the metabolic and immune levels the fluid cephalic shift disrupts the entire constituency of the blood and vascular compartment driving loss of body fluids and necessitating the cannibalizing of excess blood cells now too numerous in the reduced plasma volume, consequently launching inflammatory responses and metabolic destabilization [1,2,4,5].

Tahimic and Globus [13] note the increase in pro-inflammatory conditions, which involve immune function and general metabolism. These increases are in part the result of physiological changes enumerated in the sections above [11,12,13,14]. Blaber et al. goes further to define the system biology of space flight in a study performed on mice sent to space for only 13.5 days aboard the Space Shuttle Atlantis. This group found unmistakable evidence of increased OSaD and significant changes in the metabolism and production of glutathione signaling impairment in oxidative defense through sophisticated multi-omics enrichment analyses of metabolite and gene sets [15]. These analyses enumerated significant changes in glycerophospholipid and sphingolipid metabolism-related pathways and osmolyte concentrations possibly related to some space related dehydration. Also seen was an enrichment of purine metabolic pathways and aminoacyl-tRNA biosynthesis attendant to enrichment of autophagy associated genes and the ubiquitin-proteasome. These results, in concert with the downregulation in nuclear factor (erythroid-derived 2)-like 2-mediated signaling, suggest a decreased hepatic oxidative defense and could represent aberrant tRNA post-translational processing, induction of degradation programs and the onset of senescence-associated mitochondrial dysfunction as a consequence of the spaceflight environment [15]. Blaber and team have shown short term exposure to the space environment results in elevated ROS, OSaD, and impaired oxidative defense via suppression of NRF2-related pathways in the mouse liver. Over the long term this raises significant concern regarding potential liver damage for astronauts by way of autophagy and systemic immune related inflammation.

Anselm and colleagues followed up on the excellent work of Blaber and team by assessing metabolic pathways and conditions of mouse liver physiology after 30 days of flight on the Russian Bion-M1 spacecraft. After 30 days in space one cohort of the mice were allowed a 7-day re-adaptation while the remainder were not. Analyses of the liver proteomic profiling included shotgun mass spectrometry and label free quantification. The analyses yielded 1086 known proteins and 12,206 unique peptides and statistical testing by ANOVA revealed 218 up-regulated and 224 down-regulated proteins in the post-flight compared to the other groups [16]. Amino acid metabolism related proteins exhibited increased levels after re-adaptation, a possible indicator of elevated gluconeogenesis. In comparison to the non-adaptive flight group, mice allowed to re-adapt demonstrated reduced lipotoxicity marked by normalized levels of the peroxisome proliferator-activated receptor pathway family and bile acid secretion was normalized as a possible consequence of increased levels of transmembrane and CYP superfamily proteins during the recovery. In the non-adapted group however proteomic analyses indicated lipotoxicity, a sign of impaired fatty acid metabolism, along with altered bile secretions and glucose-uptake. These data provide a window into the advent of possible adaptive countermeasures to be used for space flight crews [16].

Investigators Burns and Manda address the vast complexities of the tricarboxylic acid (TCA) cycle and oxidative phosphorylation (OXPHOS) metabolism as inferential of OSaD in their review through an in depth description of the Warburg effect (WE), originally described to yield insights into aberrant cancer metabolism which include features of increased cellular glycolysis and remarkable changes to mitochondrial function and mtDNA repair now known to be associated with other human disease states [17]. Of note, the review illuminates the present understanding of WE regarding global diseases, cancer, diabetes, and, of particular interest, implications for spaceflight and aging. WE may be regulated through a series of events such as chain oncogenic occurrences, chosen responses consequential of restricted or impaired glucose metabolism and finally by the chance manifestation of genetic changes reflective of aging. These chain, choice or chance algorithms may be theoretically extrapolated to interpret the neurodegenerative state, as presents in Alzheimer’s and other metabolic diseases with cues to evolving therapeutics. WE pathways investigated in hostile environments with prolonged exposure like space habitation could enlighten medical countermeasures valuable in space and on Earth [17]. For extended missions to Gateway or to Mars, astronauts will require systemic countermeasures to abrogate radiation and microgravity induced OSaD and inflammation mediated by the NFκβ pathway. Representative of this might be to employ cytoprotective agents like Amifostine that initiates a metabolic shift to induction of glycolysis and blockage of mitochondrial pyruvate which can provide radioprotection to normal tissues. Also activators of the Nrf2 pathway like dimethyl fumarate and sulforaphane, capable of enhancing the endogenous antioxidant protection against ROS and RNS, are potential candidates for investigation [17].

As we turn to countermeasures we see that in many of the previous manuscripts and reviews cited potential mitigating agents are either physiologically endemic, naturally occurring, or pharmacologic [12,13,14,15,16,17]. In this review Endesfelder et al. details the mechanisms of caffeine a potent free radical scavenger and adenosine receptor antagonist. Caffeine has been shown to lower the severity of brain damage in preterm infants and has been evaluated in the research arena regarding effects on apoptosis, redox sensitive transcription factors, OSaD markers, the anti-oxidative response, inflammation and extracellular matrix in neonatal rats subjected to hyperoxia [18]. Six-day-old rats are a good model of the human fetal brain at 28–32 weeks of gestation and thus excellent for the study of OSaD and neuroprotection in the developing human brain. Rats pretreated with a single caffeine treatment demonstrated diminished OSaD markers including (glutamate-cysteine ligase catalytic subunit (GCLC hydrogen peroxide, heme oxygenase-1, and lipid peroxidation)) while promoting anti-OSaD molecules (sulfiredoxin 1, SOD and peroxiredoxin 1). Caffeine also modulated redox-sensitive transcription factor expression (Nrf2/Keap1, and NFκB) suppressed extracellular matrix degeneration (matrix metalloproteinases (MMP) 2, and inhibitor of metalloproteinase (TIMP) 1/2) and down-regulated pro-inflammatory cytokines, reduced pro-apoptotic effectors (poly (ADP-ribose) polymerase-1 (PARP-1), apoptosis inducing factor (AIF), and caspase-3) thus demonstrating caffeine to be a pleomorphic neuro-protective agent [18]. These experiments are particularly relevant to EVA and planetary exploration where astronauts will breathe ~100% O_2_ during spacesuit operations and be exposed to mild hyperoxia about 50% of the mission length overall [3,4]. 

The prospects for robust OSaD countermeasures to combat elevated radiation and microgravity effects are essential for planning long duration space missions. Velalopoulou et al. detail in their publication the prospects for the use of LGM2605, a synthetic derivative of the flaxseed lignan secoisolariciresinol diglucoside (SDG), which has been proven to reduce OSaD-related biomarkers in the presence of radiation [19]. LGM2605 was tested to determine its ability to protect and nullify the harmful effects of proton radiation (which makes up 85% of the constituents of GCR) on human lung slices. In an ex vivo model of human lung, precision-cut lung sections (huPCLS) were subjected to severe tissue toxicity via exposure to 4.0 Gy of proton radiation, having first been treated with LGM2605 then analyzed at 30 min and 24 h. post-exposure. All post exposure samples were surveyed for gene expression changes relevant to inflammation, OSaD, and cell cycle arrest. The researchers determined radiation-induced senescence, oxidative tissue damage associated cell cycle changes, and an associated proinflammatory phenotype in non-treated samples. To summarize, the data provides evidence of the significant protective capability by LGM2605. Additionally, LGM2605-pretreatment of proton-irradiated huPCLS significantly upregulates anti-OSaD genes and protects huPCLS from a senescent-like phenotype, regulated at the gene and protein level by p53, members of the cyclin-dependent kinase (CDK) family, and p21. LGM2506 pretreatment substantially reduced p16 induced by proton radiation, plus downregulates proinflammatory cytokine gene levels [19]. Downstream protective effects LGM2506 may yet be realized and may be an excellent candidate for astronaut protections from radiation, microgravity effects and respiratory exposure to hyperoxia and hypoxia. 

In conclusion, the 10 significant and elegant manuscripts published in this Special Issue illustrate the intricacies, relevance, and impacts of unstable ROS generation and persistent OSaD in the space flight environment, which are, in many ways, emblematic of the phenomena of aging. Recent data released by NASA on the human Twins Study further illustrates the changes to and potential damage sustained by the human physiology while inhabiting an unrelentingly hostile environment, especially considering the results of analyses of inflammatory cytokines, RNA and DNA analyses and microbiome research conducted by Drs. Snyder, Mason, and Turek, respectively [21]. We would like to thank each of the authors contributing to this Special Issue, for their insights, talents, and enduring interest in furthering the advancement of humankind’s quest to travel to and one day inhabit far-off worlds. This arena of research also serves a remarkably timely application, namely that of potentially understanding and ameliorating the myriad of diseases associated with the inevitable process of aging.

## Figures and Tables

**Table 1:** Contributions to the Special Issue “Oxidative Stress and Space Biology: An Organ-Based Approach.

Authors	Title	Topic	Type
Alwood et al.	Dose- and Ion-Dependent Effects in the Oxidative Stress Response to Space-Like Radiation Exposure in the Skeletal System	Bone	Original Research
Shanmugarajan et al.	Combined Effects of Simulated Microgravity and Radiation Exposure on Osteoclast Cell Fusion	Bone/Protective	Original Research
Tian et al.	The Impact of Oxidative Stress on the Bone System in Response to the Space Special Environment	Bone	Review
Tahimic et al.	Redox Signaling and Its Impact on Skeletal and Vascular Responses to Spaceflight	Bone/Cardio	Review
Takahashi et al.	Effect of Oxidative Stress on Cardiovascular System in Response to Gravity	Cardiovascular	Review
Blaber et al.	Spaceflight Activates Autophagy Programs and the Proteasome in Mouse Liver	Immune/Metabolism	Original Article
Anslem et al.	Re-adaption on Earth after Spaceflights Affects the Mouse Liver Proteome	Immune/Metabolism	Original Article
Burns and Manda	Metabolic Pathways of the Warburg Effect in Health and Disease: Perspectives of Choice, Chain or Chance	Immune/Metabolism	Review
Endesfelder et al.	Neuroprotection by Caffeine in Hyperoxia-Induced Neonatal Brain Injury	Neuro/Protective	Original Article
Velalopoulou et al.	Synthetic Secoisolariciresinol Diglucoside (LGM2605) Protects Human Lung in an Ex Vivo Model of Proton Radiation Damage	Lung/Protective	Original Article

## Abbreviations

BLEO	Beyond Low Earth Orbit
CDK	Cyclin-Dependent Kinase
DNA	Deoxyribonucleic Acid
EVA/IVA	Extravehicular Activity and Intra-Vehicular Activity
HLU	Hind Limb Unloading
GCR	Galactic Cosmic Radiation
GPx-1	Glutathione Peroxidase
cGy	Centi Gray
Gy	Gray
H_2_O_2_	Hydrogen Peroxide
LEO	Low Earth Orbit
LDR	Low Dose Radiation
Mn-SOD	Manganese Superoxide Dismutase
NO	Nitric Oxide
NOX_2_	Nicotinamide Adenine Dinucleotide Phosphate-oxidase (isoform 2)
NOX_4_	Nicotinamide Adenine Dinucleotide Phosphate-oxidase (isoform 4)
eNOS	Endothelial Nitric Oxide Synthase
OSaD	Oxidative Stress and Damage
OXPHOS	Oxidative Phosphorylation
RAW 264.7	Murine Monocyte Cell Line Identification
REDOX	Reduction and Oxidation
RNA	Ribonucleic Acid
ROS/RNS	Reactive Oxygen and Reactive Nitrogen Species
SDG	Flaxseed Lignan Secoisolariciresinol Diglucoside
SOD	Super Oxide Dismutase
TCA	Tricarboxcylic Acid
Wnt	Wingless-Type MMTV (mouse mammary tumor virus)
